# Early to mid-pregnancy HbA1c levels and its association with adverse pregnancy outcomes in three low middle-income countries in Asia and Sub-Saharan Africa

**DOI:** 10.1186/s12884-023-06241-w

**Published:** 2024-01-15

**Authors:** Muhammad Imran Nisar, Sayan das, Rasheda Khanam, Javairia Khalid, Swagata Chetia, Tarik Hasan, Shahira Shahid, Msafiri Ladislaus Marijani, Salahuddin Ahmed, Farah Khalid, Said Mohammed Ali, Nabidul Haque Chowdhury, Usma Mehmood, Arup Dutta, Sayedur Rahman, Muhammad Farrukh Qazi, Saikat Deb, Dipak Kumar Mitra, Asra Abeer Usmani, Usha Dhingra, Rubhana Raqib, Alexander Manu, Sachiyo Yoshida, Nicole Minckas, Rajiv Bahl, Abdullah H. Baqui, Sunil Sazawal, Fyezah Jehan

**Affiliations:** 1 Department of Pediatrics and Child Health, The Aga Khan University, Stadium Road, Karachi, 74800 Pakistan; 2 Center for Public Health Kinetics, New Delhi, India; 3 Department of International Health, Johns Hopkins Bloomberg School of Public Health, Maryland, Baltimore, USA; 4 Projahnmo Research Foundation, Dhaka, Bangladesh; 5 Ministry of Health, Zanzibar, Tanzania; 6 Public Health Laboratory-IDC, Pemba, Tanzania; 7 Department of Women’s and Children’s Health, Uppsala University, Uppsala, Sweden; 8 Department of Public Health, School of Health and Life Sciences, North South University, Dhaka, Bangladesh; 9 International Center for Diarrheal Disease Research, Dhaka, Bangladesh; 10 London School of Hygiene & Tropical Medicine Faculty of Epidemiology and Public Health, London, UK; 11 Department for Maternal, Child, Adolescents and Ageing Health, World Health Organization (MCA/MRD), Avenue Appia 20, Geneva, 1211 Switzerland

**Keywords:** HbA1c, First trimester, Gestational Diabetes Mellitus, Adverse pregnancy outcomes, South Asia, Sub-Saharan Africa, LMICs

## Abstract

**Background:**

Hyperglycemia during pregnancy leads to adverse maternal and fetal outcomes. Thus, strict monitoring of blood glucose levels is warranted. This study aims to determine the association of early to mid-pregnancy HbA1c levels with the development of pregnancy complications in women from three countries in South Asia and Sub-Saharan Africa.

**Methods:**

We performed a secondary analysis of the AMANHI (Alliance for Maternal and Newborn Health Improvement) cohort, which enrolled 10,001 pregnant women between May 2014 and June 2018 across Sylhet-Bangladesh, Karachi-Pakistan, and Pemba Island-Tanzania. HbA1c assays were performed at enrollment (8 to < 20 gestational weeks), and epidemiological data were collected during 2–3 monthly household visits. The women were followed-up till the postpartum period to determine the pregnancy outcomes. Multivariable logistic regression models assessed the association between elevated HbA1c levels and adverse events while controlling for potential confounders.

**Results:**

A total of 9,510 pregnant women were included in the analysis. The mean HbA1c level at enrollment was found to be the highest in Bangladesh (5.31 ± 0.37), followed by Tanzania (5.22 ± 0.49) and then Pakistan (5.07 ± 0.58). We report 339 stillbirths and 9,039 live births. Among the live births were 892 preterm births, 892 deliveries via cesarean section, and 532 LGA babies. In the multivariate pooled analysis, maternal HbA1c levels of ≥ 6.5 were associated with increased risks of stillbirths (aRR = 6.3, 95% CI = 3.4,11.6); preterm births (aRR = 3.5, 95% CI = 1.8–6.7); and Large for Gestational Age (aRR = 5.5, 95% CI = 2.9–10.6).

**Conclusion:**

Maternal HbA1c level is an independent risk factor for predicting adverse pregnancy outcomes such as stillbirth, preterm birth, and LGA among women in South Asia and Sub-Saharan Africa. These groups may benefit from early interventional strategies.

**Supplementary Information:**

The online version contains supplementary material available at 10.1186/s12884-023-06241-w.

## Background

Hyperglycemia in pregnancy affected around 21.1 million (16.7%) live births in 2021. The majority (80.3%) of these were diagnosed for the first time during pregnancy and went on to be classified as Gestational Diabetes Mellitus (GDM) [1]. The burden is disproportionately higher in low- and middle-income countries (LMICs) where socioeconomic and environmental stressors such as exposure to poor nutrition in early childhood, limited access to healthcare facilities, and a genetic predisposition in certain ethnicities are thought to contribute to the higher burden [2, 3]. Several studies have shown a linear relationship between blood glucose levels during pregnancy with adverse maternal–fetal outcomes and the risk of diabetes mellitus later in life [4]. Increased placental transport of glucose leads to elevated fetal insulin and insulin-like growth factor 1 (IGF-1) levels causing fetal overgrowth or macrosomia [5]. Macrosomia increases the risk of obstructed labor and cesarean delivery [6]. Excess fetal insulin production can contribute to β-cell dysfunction and insulin resistance, increasing the risk of hypoglycemia and brain injury after birth [7]. There is also increased risk of stillbirth and preterm births, due to mechanisms such as oxidative stress, placental dysfunction, pre-eclampsia, and fetal macrosomia [8]. Thus, it is important to screen women for elevated glucose levels to prevent serious complications of pregnancy. The American Diabetes Association (ADA) has recommended fasting blood glucose levels and oral glucose tolerance test (OGTT) as the gold standard diagnostic tests for GDM [9]. However, both of these tests require prolonged fasting and the Oral Glucose Tolerance Test (OGTT) can be practically burdensome in low-resource settings with limited access to healthcare. In contrast, the glycated hemoglobin (HbA1c) is a test which gives average glucose levels during the preceding 90–120 days. It is routinely used for monitoring glycemic control in diabetic patients. An HbA1c percent greater than 6.5 is diagnostic of diabetes mellitus in non-pregnant individuals [10]. In addition, if performed as a point-of-care test, it can improve testing compliance for monitoring hyperglycemia in a single visit. However, there is currently no clear consensus on its use in the screening and management of pregnant women for GDM.

Thus, in this paper, we refer to our experience of performing point of care HbA1c testing as a biomarker of hyperglycemia during early to mid-pregnancy on a large cohort of pregnant women across three countries in Asia and Africa and its association with adverse pregnancy outcomes like stillbirth, preterm birth, large for gestational age and cesarean section [11].

## Methods

### Study design and setting

We performed a secondary data analysis on a large cohort of pregnant women enrolled as part of the Alliance for Maternal and Child Health Improvement (AMANHI) biorepository study. Between May 2014 and June 2018, the AMANHI study enrolled 10,001 pregnant women between 8—< 20 weeks of gestational age from Bangladesh, Pakistan, and Tanzania. A detailed description of the study sites and characteristics of the cohort has been published previously [11]. Briefly, women were enrolled after confirming pregnancy and gestational age through ultrasound scan, and blood and urine samples were collected using standardized methods across the three sites at the time of enrollment, 24–28 or 32–36 weeks of gestation, at the time of birth and 6 weeks after delivery. Placental tissue and maternal and newborn stool samples were also collected at the time of birth. In addition, a paternal saliva sample was collected. At each contact, trained field workers collected detailed information on the health and care seeking behavior of the pregnant woman using a standardized tool across all sites [11].

For HbA1c testing, trained phlebotomists collected 0.25–0.50 ml of maternal venous blood in a purple top 7.5 ml EDTA tube (S-Monovette). HbA1c level was measured via a monoclonal antibody agglutination reaction using the Siemens DCA Vantage® Analyzer (Siemens, Washington, USA), with controls traceable to the International Federation of Clinical Chemistry (IFCC) reference materials and test methods for measurement of HbA1c. The normal range of this HbA1c level measurement was within 2.5% to 14% units (4 mmol/mol to 130 mmol/mol) according to the manufacturer.

### Statistical analysis

The primary exposure variable for this analysis was HbA1c levels measured at enrollment (8 to < 20 weeks of gestation) and categorized based on the ADA guidelines into: less than 5.7%, 5.7–6.4% and ≥ 6.5% [12]. For the outcomes of interest, stillbirths were defined as babies who were born dead after 22 weeks of gestation. Among livebirths, preterm births were defined as livebirths before 37 weeks of gestation; large for gestational age (LGA) births were defined as liveborn with a birthweight above 90th percentile based on INTERGROWTH-21st standards [13]. Mid-upper arm circumference (MUAC) was categorized as severely malnourished < 21 cm, moderately malnourished ≥ 21 cm & < 23 cm, and normal ≥ 23 cm. Body Mass Index (BMI) was categorized as underweight < 18.5, normal ≥ 18.5 & < 25.0, overweight ≥ 25.0and obese ≥ 30.0. The fourth outcome was children born through cesarean section.

For descriptive purposes, all continuous variables were expressed as mean ± SD and categorical variables as frequencies with percentages. Generalized binomial regression was used to estimate crude and adjusted risk ratios for HbA1c levels with the four predefined outcomes. We used stepwise regression with forward selection. The final multivariate model had all variables with a p-value less than 0.05. The model was adjusted for the following covariates: maternal age; education status; wealth quintile; parity; gravidity; MUAC; BMI; smoking and tobacco use; exposure to biomass; history of previous stillbirths, miscarriages, caesarean section, and preterm birth; hypertension; diabetes; anemia; gender of the fetus; place and mode of delivery; and history of antepartum and postpartum hemorrhage. Women with missing HbA1c levels at enrollment, multiple births, abortive outcomes, and those missing outcome information were excluded from the analyses. All analysis was performed using Stata version 15.0.

### Patient and public involvement

It was not appropriate or possible to involve patients or the public in the design, conduct, or reporting, or dissemination plans of our research.

### Ethics

The AMANHI study received ethical approval from the local and institutional ethics committees of all the three sites. These included Zanzibar Health Research Ethics Committee (formerly ZAMREC) (ZAMREC/0002/OCTOBER/013) for Tanzania, ICDDR, B (PR12073) and John Hopkins University (IRB 00004508) for Bangladesh and Aga Khan University (2790-paeds-ERC-13) for Pakistan. In addition, the protocols for the biorepository study were also approved by the WHO Ethics Review Committee (RPC 532) and continuing approvals were sought yearly. Written informed consent was obtained from study participants in which all study and sample handling and study procedures were explained in detail. HbA1c results were also shared with these participants.

## Results

### Pregnancy outcomes and maternal characteristics

**Fig. 1:**
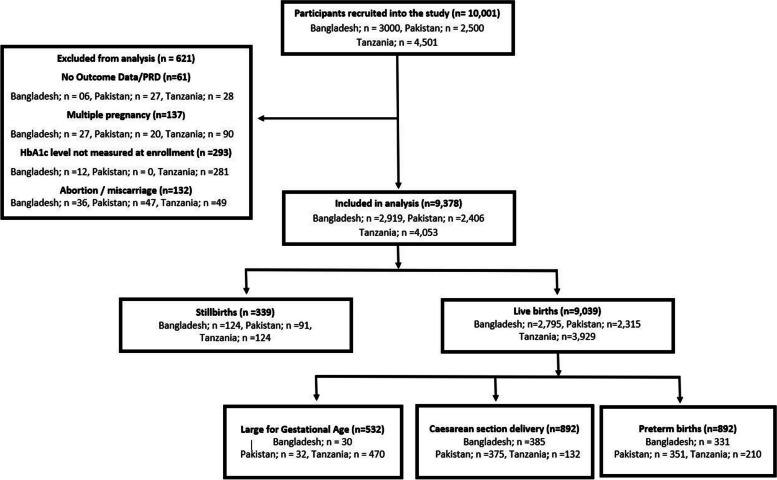
Flowchart showing recruitment of participants in the study

A total of 10,001 women were enrolled in the study across the three sites from May 2014 and June 2018. HbA1c levels at enrolment were missing for 293 pregnant women; 137 women had multifetal pregnancies; 132 pregnancies ended in abortion or a miscarriage, and outcome information was missing for 61 women. These were excluded from the analysis. The remaining pregnancies (*n* = 9,378) resulted in 9,039 liveborn babies (96.4%) and 339 stillbirths (3.6%). There were 892 preterm births (9.8%), 892 women underwent a C-Sect. (9.8%), and 532 babies were born large for gestational age (5.9%). The site-wise distribution of these pregnancy outcomes is given in Fig. 1.

Table 1 summarizes the clinical and sociodemographic characteristics of the enrolled women.

**Table 1:** Baseline characteristics for pregnancies included in the secondary analysis

**Variables**		**Sylhet-Bangladesh N (%)**	**Karachi-Pakistan N (%)**	**Pemba-Tanzania N (%)**	**Overall N (%)**
		***N* = 2,955**	***N* = 2,453**	***N* = 4,102**	***N* = 9,510**
**Socio-demographics and maternal characteristics**					
**Mother age**	< 20 Years	1,037 (35.1%)	346 (14.1%)	528 (12.9%)	1,911 (20.1%)
	20–29 Years	1,565 (53.0%)	1,407 (57.4%)	1,958 (47.7%)	4,930 (51.8%)
	30–39 Years	348 (11.8%)	672 (27.4%)	1,386 (33.8%)	2,406 (25.3%)
	40–49 Years	5 ( 0.2%)	28 ( 1.1%)	216 ( 5.3%)	249 ( 2.6%)
	No Data	0 ( 0.0%)	0 ( 0.0%)	14 ( 0.3%)	14 ( 0.1%)
	**Mean age of women ± SD**	23.5 ± 4.4	26.7 ± 5.2	27.9 ± 6.3	26.2 ± 5.8
**Mother education**	No formal education	188 ( 6.4%)	1,284 (52.3%)	552 (13.5%)	2,024 (21.3%)
	Primary	1,066 (36.1%)	413 (16.8%)	0 ( 0.0%)	1,479 (15.6%)
	Middle	821 (27.8%)	288 (11.7%)	1,439 (35.1%)	2,548 (26.8%)
	Secondary	711 (24.1%)	376 (15.3%)	2,034 (49.6%)	3,121 (32.8%)
	Graduation or higher	159 ( 5.4%)	92 ( 3.8%)	64 ( 1.6%)	315 ( 3.3%)
	No data	10 ( 0.3%)	0 ( 0.0%)	13 ( 0.3%)	23 ( 0.2%)
	**Mean year of education ± SD**	6.6 ± 2.9	3.5 ± 4.2	7.3 ± 3.5	6.1 ± 3.9
**Parity**	No previous births	142 ( 4.8%)	151 ( 6.2%)	76 ( 1.9%)	369 ( 3.9%)
	1–2 births	1,272 (43.0%)	1,075 (43.8%)	1,098 (26.8%)	3,445 (36.2%)
	3–5 births	514 (17.4%)	591 (24.1%)	1,412 (34.4%)	2,517 (26.5%)
	> 5 births	1,023 (34.6%)	636 (25.9%)	1,503 (36.6%)	3,162 (33.2%)
	No data	4 ( 0.1%)	0 ( 0.0%)	13 ( 0.3%)	17 ( 0.2%)
**Gravidity**	1	973 (32.9%)	468 (19.1%)	717 (17.5%)	2,158 (22.7%)
	2	731 (24.7%)	516 (21.0%)	385 ( 9.4%)	1,632 (17.2%)
	3	537 (18.2%)	461 (18.8%)	593 (14.5%)	1,591 (16.7%)
	≥ 4	710 (24.0%)	1,008 (41.1%)	2,394 (58.4%)	4,112 (43.2%)
	No data	4 ( 0.1%)	0 ( 0.0%)	13 ( 0.3%)	17 ( 0.2%)
**MUAC (in cm)**	Severely malnourished < 21	386 (13.1%)	219 ( 8.9%)	74 ( 1.8%)	679 ( 7.1%)
	Moderately malnourished ≥ 21 & < 23	662 (22.4%)	374 (15.2%)	287 ( 7.0%)	1,323 (13.9%)
	Normal ≥ 23	908 (30.7%)	1,335 (54.4%)	2,731 (66.6%)	4,974 (52.3%)
	No data	999 (33.8%)	525 (21.4%)	1,010 (24.6%)	2,534 (26.6%)
	**Mean MAUC ± SD**	23.0 ± 3.0	25.0 ± 3.8	28.3 ± 4.9	25.9 ± 4.7
**Mother height (cm)**	< 150	1,424 (48.2%)	537 (21.9%)	644 (15.7%)	2,605 (27.4%)
	150–164.99	1,512 (51.2%)	1,663 (67.8%)	2,805 (68.4%)	5,980 (62.9%)
	≥ 165	9 ( 0.3%)	100(4.1%)	201 ( 4.9%)	310( 3.3%)
	no data	10 ( 0.3%)	153( 6.2%)	452 (11.0%)	615( 6.5%)
	**Mean height ± SD**	149.6 ± 5.4	153.7 ± 6.4	155.3 ± 6.3	153 ± 6.5
**BMI**	Underweight < 18.50	957 (32.4%)	504 (20.6%)	237 ( 5.8%)	1,698 (17.9%)
	Normal ≥ 18.50 & < 25.00	1,773 (60.0%)	1,187 (48.4%)	1,948 (47.5%)	4,908 (51.6%)
	Overweight ≥ 25.00	161 ( 5.4%)	435 (17.7%)	871 (21.2%)	1,467 (15.4%)
	Obese ≥ 30.00	31 ( 1.0%)	171 ( 7.0%)	589 (14.4%)	791 ( 8.3%)
	No data	33 ( 1.1%)	156 ( 6.4%)	457 (11.1%)	646 ( 6.8%)
	**Mean BMI ± SD**	20.0 ± 3.1	22.3 ± 4.8	24.7 ± 5.3	22.6 ± 4.9
**Smoking & tobacco use**		499 (16.9%)	498 (20.3%)	28 ( 0.7%)	1,025 (11.1%)
**Exposure to biomass**		2,870 (97.2%)	268 (10.9%)	3,461 (89.6%)	6,599 (71.2%)
**History of past pregnancies**					
**Stillbirth**		232 ( 7.9%)	153 ( 6.2%)	272 ( 6.6%)	657 ( 6.9%)
**Miscarriage**		380 (12.9%)	735 (30.0%)	1,105 (26.9%)	2,220 (23.3%)
**Caesarean section**		59 ( 2.0%)	186 ( 7.6%)	106 ( 2.7%)	351 ( 3.8%)
**Pre**term** birth**		125 ( 4.2%)	175 ( 8.8%)	84 ( 2.3%)	384 ( 4.5%)
**Hypertension**		6 ( 0.2%)	142 (5.8%)	113 (2.9%)	261 ( 2.8%)
**Diabetes**		10 ( 0.3%)	19 ( 0.8%)	20 ( 0.5%)	49 ( 0.5%)
**Current pregnancies**					
**Gender of fetus**	Male	1,365 (46.2%)	1,145 (46.7%)	1,911 (46.6%)	4,421 (46.5%)
	Female	1,430 (48.4%)	1,080 (44.0%)	1,825 (44.5%)	4,335 (45.6%)
	No Data	160 ( 5.4%)	228 ( 9.3%)	366 ( 8.9%)	754 ( 7.9%)
**Place of delivery**	Health Facility	1,985(67.2%)	1,634(66.6%)	3,241(79.0%)	6,860(72.1%)
	Home	746(25.2%)	708(28.9%)	661(16.1%)	2551(22.2%)
	No Data	224(7.6%)	111(4.5%)	200(4.9%)	535(5.6%)
**Mode of delivery**	Vaginal	2,509 (84.9%)	1,862 (75.9%)	3,748 (91.4%)	8,119 (85.4%)
	Vaginal assisted (e.g., forceps, vacuum)	4 ( 0.1%)	54 ( 2.2%)	10 ( 0.2%)	68 ( 0.7%)
	Caesarean section	395 (13.4%)	386 (15.7%)	144 ( 3.5%)	925 ( 9.7%)
	No data	47 ( 1.6%)	151 ( 6.2%)	200 ( 4.9%)	398 ( 4.2%)
**Severe antepartum infection**		49(1.8%)	527(22.8%)	21(0.6%)	597(6.8%)
**Severe antepartum hemorrhage**		41(1.4%)	288(12.0)	155(3.8)	484(5.1%)
**Severe postpartum infection**		92 ( 3.4%)	293 (12.7%)	130 ( 3.5%)	515 ( 5.9%)
**Severe postpartum hemorrhage**		37 ( 1.4%)	32 ( 1.4%)	2,378 (63.9%)	2,447 (27.9%)

Most women were in the 20–29 years age bracket with the lowest mean maternal age found in Bangladesh (23.46 ± 4.44 years). In total 679 (7.1%) women were severely malnourished and 1323 (13%) were moderately malnourished, with the highest percentage of malnourished women in Bangladesh. Pakistan site had the highest proportion of women who had no formal education (52%, *n* = 1284). History of miscarriage in a previous pregnancy was also highest in Pakistan (30%, *n* = 735).

Mean HbA1c levels at enrollment for the whole cohort was 5.2% (± 0.5%). It was highest for Bangladesh (5.31 ± 0.37), followed by Tanzania (5.22 ± 0.49) and then Pakistan (5.07 ± 0.58) (Fig. S1). Using the ADA cutoff values for diabetes mellitus, 8486 number of women (89%) had HbA1c levels below 5.7%, 946 (10%) had HbA1c levels between 5.7 -6.4, and 78 (1%) were above ≥ 6.5%. Tanzania site had the highest number of women 42 (6.5%) with HbA1c levels more than 6.5 (Table 2).

**Table 2:** Pregnancy outcomes and HbA1c levels of the enrolled women

**Outcomes**	**Sylhet-Bangladesh** ***N* = 2955**		**Karachi-Pakistan** ***N* = 2453**		**Pemba-Tanzania** ***N* = 4102**		**Total** ***N* = 9510**	
	**Yes**	**No**	**Yes**	**No**	**Yes**	**No**	**Yes**	**No**
**Stillbirth** **Gestational Age > 22 Weeks)**	*N* = 124	*N* = 2831	*N* = 91	*N* = 2362	*N* = 124	*N* = 3978	*N* = 339	*N* = 9171
**HbA1c < 5.7**	102 (82.3)	2515 (88.8)	81 (89.9)	2253 (95.4)	103 (86.1)	3432 (86.3)	286 (84.4)	8200 (89.4)
**HbA1c 5.7–6.4**	20 (16.1)	304 (10.7)	5 (5.5)	92 (3.9)	13 (10.5)	512 (12.9)	38 (11.2)	908 (9.9)
**HbA1c ≥ 6.5**	2 (1.6)	12 (0.4)	5 (5.5)	17 (0.7)	8 (6.4)	34 (0.8)	15 (4.4)	63 (0.7)
**Preterm birth** **(< 37 weeks of Gestation)**	*N* = 331	*N* = 2464	*N* = 351	*N* = 1961	*N* = 210	*N* = 3604	*N* = 892	*N* = 8029
**HbA1c < 5.7**	288 (87.0)	2194 (89.0)	327 (93.2)	1879 (95.8)	175 (83.3)	3116 (86.5)	790 (88.6)	7189 (89.5)
**HbA1c 5.7–6.4**	38 (11.5)	263 (10.7)	19 (5.4)	71 (3.6)	29 (13.8)	461 (12.8)	86 (9.6)	795 (9.9)
**HbA1c ≥ 6.5**	5 (1.5)	7 (0.3)	5 (1.4)	11 (0.6)	6 (2.9)	27 (0.7)	16 (1.8)	45 (0.6)
**Large for gestational age** **(> 10th centile)**	*N* = 30	*N* = 2362	*N* = 32	*N* = 1943	*N* = 470	*N* = 3167	*N* = 532	*N* = 7472
**HbA1c < 5.7**	25 (83.3)	2099 (88.9)	27 (84.4)	1858 (95.6)	382 (81.3)	2747 (86.7)	434 (81.6)	6704 (89.7)
**HbA1c 5.7–6.4**	2 (6.7)	255 (10.8)	2 (6.3)	75 (3.9)	74 (15.7)	403 (12.7)	78 (14.6)	733 (9.8)
**HbA1c ≥ 6.5**	3 (10.0)	8 (0.3)	3 (9.3)	10 (0.5)	14 (3.0)	17 (0.5)	20 (3.8)	35 (0.5)
**Cesarean section delivery**	*N* = 385	*N* = 2410	*N* = 375	*N* = 1815	*N* = 132	*N* = 3674	*N* = 892	*N* = 7899
**HbA1c < 5.7**	339 (88.0)	2143 (88.9)	346 (92.3)	1742 (96.0)	108 (81.8)	3175 (86.4)	793 (88.9)	7060 (89.4)
**HbA1c 5.7–6.4**	43 (11.2)	258 (10.7)	21 (5.6)	65 (3.6)	22 (16.7)	468 (12.7)	86 (9.6)	791 (10.0)
**HbA1c ≥ 6.5**	3 (0.8)	9 (0.4)	8 (2.1)	8 (0.4)	2 (1.5)	31 (0.9)	13 (1.5)	48 (0.6)

Figure S2a, b and c show the HbA1c levels by categories of maternal Age, BMI and MUAC across all study sites.

### Association of HbA1c levels with adverse pregnancy outcomes

**Fig. 2:**
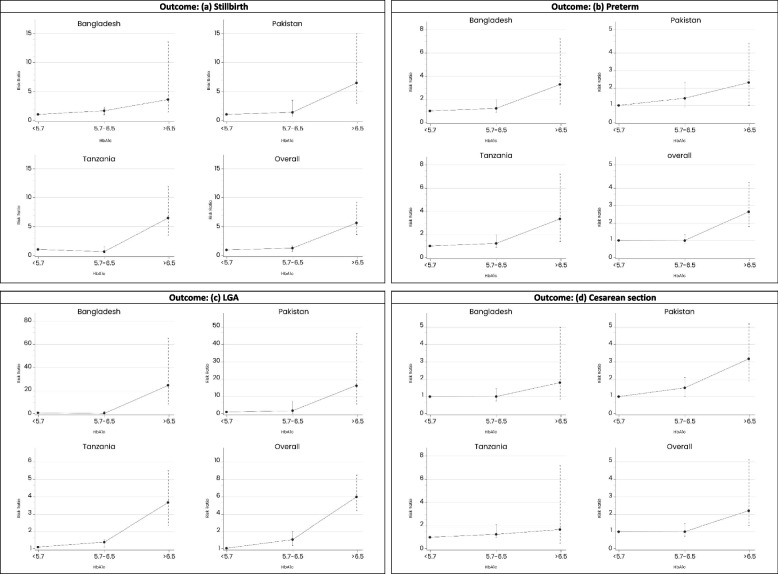
Site-specific risk ratios of HbA1c categories with adverse pregnancy outcomes (**a**) Stillbirth, (**b**) preterm, (**c**) LGA and (**d**) c-section

Table 3 and Fig. 2 (a,b,c and d) shows the association between HbA1c levels at less than 20 gestational weeks and adverse pregnancy outcomes across all sites. In the unadjusted model, HbA1c levels ≥ 6.5 were found to be significantly associated with stillbirths (RR = 5.7, 95% CI 3.6,9.1), preterm births (RR = 2.6, 95%CI 1.7,4.1), LGA (RR = 6.0, 95%CI 4.2, 8.6) and C-section deliveries (RR = 2.1, 95%CI 1.3,5.3).

**Table 3:** Pooled and site-wise univariate analysis for adverse birth outcomes according to Hba1c levels

**Outcomes**	**Sylhet-Bangladesh** ***N* = 2955**	**Karachi-Pakistan** ***N* = 2453**	**Pemba-Tanzania** ***N* = 4102**	**Total** ***N* = 9510**
	**RR(95%CI)**	**RR(95%CI)**	**RR(95%CI)**	**RR(95%CI)**
**Pregnancy ended in stillbirth (Gestational Age > 22 Weeks)**	***N* = 124**	***N* = 91**	***N* = 124**	***N* = 339**
HbA1c < 5.7	Ref	Ref	Ref	Ref
HbA1c 5.7–6.4	1.6 (1.0–2.2)	1.5(0.6–3.6)	0.8(0.5–1.5)	1.2(0.9–1.7)
HbA1c ≥ 6.5	3.7 (1.6–13.4)	6.5(2.9–14.6)	6.5(3.4–12.5)	5.7(3.6–9.1)
**Preterm birth** **(< 37 weeks of Gestation)**	***N* = 331**	***N* = 351**	***N* = 210**	***N* = 892**
HbA1c < 5.7	Ref	Ref	Ref	Ref
HbA1c 5.7–6.4	1.1 (0.8–1.5)	1.4 (0.9–2.1)	1.1 (0.8–1.6)	1.0 (0.8–1.2)
HbA1c ≥ 6.5	3.6 (1.8–7.1)	2.1 (1.0–4.4)	3.4 (1.6–7.1)	2.6 (1.7–4.1)
**Large for gestational age** **(> 10th centile)**	***N* = 30**	***N* = 32**	***N* = 470**	***N* = 532**
HbA1c < 5.7	Ref	Ref	Ref	Ref
HbA1c 5.7–6.4	0.7 (0.2–2.8)	1.8 (0.4–7.5)	1.3 (1.0–1.6)	1.6 (1.3–2.0)
HbA1c ≥ 6.5	23.1 (8.2–65.6)	16.1 (5.6–46.5)	3.7 (2.5–5.5)	6.0 (4.2–8.6)
**Caesarean section delivery**	***N* = 385**	***N* = 375**	***N* = 132**	***N* = 892**
HbA1c < 5.7	Ref	Ref	Ref	Ref
HbA1c 5.7–6.4	1.0 (0.8–1.4)	1.5 (1.0–2.2)	1.4 (0.9–2.2)	1.0 (0.8–1.2)
HbA1c ≥ 6.5	1.8 (0.7–5.0)	3.2 (1.9–5.3)	1.9 (0.5–7.4)	2.1 (1.3–5.3)

In the multivariate analysis, the adjusted relative risks (aRR) for HbA1c levels 5.7–6.4 were (aRR = 1.2, 95% CI 0.9, 1.7) for stillbirths; (aRR = 1.2, 95% CI 0.9, 1.6) for preterm births; and (aRR = 1.2, 95% CI 0.9, 1.6) for LGA. For HbA1c levels ≥ 6.5, the aRR was (aRR = 6.3, 95% CI 3.4, 11.6) for stillbirths; (aRR = 3.5, 95% CI 1.8, 6.7) for preterm births; and (aRR = 5.5, 95% CI 2.9, 10.6) for LGA. (Table 4).

**Table 4:** Pooled and site-wise adjusted analysis for adverse birth outcomes according to Hba1c levels

**Outcomes**	**Sylhet-Bangladesh** ***N* = 2955**	**Karachi-Pakistan** ***N* = 2453**	**Pemba-Tanzania** ***N* = 4102**	**Total** ***N* = 9510**
	**aRR(95%CI)**	**aRR(95%CI)**	**aRR(95%CI)**	**aRR(95%CI)**
**Pregnancy ended in stillbirth (Gestational Age > 22 Weeks)**	***N* = 124**	***N* = 91**	***N* = 124**	***N* = 339**
HbA1c < 5.7	-	Ref	Ref	Ref
HbA1c 5.7–6.4	-	1.5 (0.6–3.9)	0.5 (0.2–1.2)	1.2 (0.9–1.7)
HbA1c ≥ 6.5	-	10.1 (3.6–28.4)	8.3 (2.8–24.3)	6.3 (3.4–11.6)
**Preterm birth** **(< 37 weeks of Gestation)**	***N* = 331**	***N* = 351**	***N* = 210**	***N* = 892**
HbA1c < 5.7	Ref	-	-	Ref
HbA1c 5.7–6.4	1.2 (0.8–1.9)	-	-	1.2 (0.9–1.6)
HbA1c ≥ 6.5	6.8 (1.8–25.9)	-	-	3.5 (1.8–6.7)
**Large for gestational age** **(> 10th centile)**	***N* = 30**	***N* = 32**	***N* = 470**	***N* = 532**
HbA1c < 5.7	Ref	Ref	Ref	Ref
HbA1c 5.7–6.4	0.5 (0.1–2.4)	1.4 (0.3–6.1)	1.1 (0.8–1.4)	1.2 (0.9–1.6)
HbA1c ≥ 6.5	34.8 (8.8–139.6)	12.0 (2.8–52.0)	4.1 (1.9–8.4)	5.5 (2.9–10.6)
**Caesarean section delivery**	***N* = 385**	***N* = 375**	***N* = 132**	***N* = 892**
HbA1c < 5.7	-	Ref	-	-
HbA1c 5.7–6.4	-	1.3 (0.7–2.2)	-	-
HbA1c ≥ 6.5	-	5.7 (1.8–18.3)	-	-

The stillbirth model was adjusted for gravidity, previous history of stillbirth, tobacco use, biomass exposure and maternal anemia. The preterm model was adjusted for maternal age, MUAC, parity, gravidity, previous cesarean section, tobacco use and biomass exposure. The LGA model was adjusted for maternal education status, maternal BMI, MUAC, gravidity, biomass exposure, tobacco use, and previous history of diabetes. The cesarean section model was adjusted for maternal age, wealth quintile, maternal BMI, parity, and gravidity

## Discussion

In our study, maternal HbA1c levels during early to mid- pregnancy (8 to < 20 gestational weeks) were associated with stillbirth, preterm birth, and LGA deliveries in Bangladesh, Pakistan and Tanzania. The majority of hyperglycemia during pregnancy remains undiagnosed in sub-Saharan Africa and South Asia [14]. Only half of the pregnant women in these regions receive the minimum recommended four antenatal care visits and most of the births occur at home, typically attended by traditional birth attendants who lack skills to manage the complications of hyperglycemia in pregnancy [15]. In this situation, point-of-care HbA1c testing during early to mid-pregnancy can serve as an optimal biomarker for identifying women at an increased risk of adverse outcomes. The ADA has previously suggested that HbA1c levels below 6.0% (42 mmol/mol) in mid-pregnancy are associated with the lowest risk of maternal complications [16]. In our study, 97.1% of the women had HbA1c levels below this cutoff, suggesting that women above this cutoff could be predisposed to adverse outcomes. We also found the ADA defined category of 6.5 and above for the diagnosis of diabetes mellitus in non-pregnant population to be associated with a higher risk of adverse pregnancy outcomes. Thus, a target of < 5.7% could be optimal during pregnancy in our population, provided it can be achieved without significant hypoglycemia.

There have been several previous attempts to define an optimal HbA1c cut-off for predicting adverse pregnancy outcomes in healthy pregnant women without pre-existing diabetes. The HAPO Study (Hyperglycemia and Adverse Pregnancy Outcomes), enrolled 5000 women across the globe and found that higher HbA1c levels at 24–32 weeks of gestation were associated with an increased risk of primary cesarean delivery, neonatal hypoglycemia, and large-for-gestational-age infants [17]. A longitudinal study from New Zealand conducted by Hughes et al. including 16,122 women predominantly of non-Hispanic white origin, demonstrated that a first trimester HbA1c threshold of 5.9% was associated with an increased risk of adverse pregnancy outcomes, including major congenital anomaly, preeclampsia, perinatal death, large for gestational age and preterm birth [18]. This cut-off is also lower than the ADA-defined cut-off of ≥ 6.0%. Bender et al., reported a cut-off of ≥ 5.7% at the first prenatal visit which could be used to identify women at increased risk for adverse pregnancy outcomes, including preterm birth, small for gestational age infants, and admission to the neonatal intensive care unit [19]. In addition, Antoniou et al. proposed an even lower cut-off of ≥ 5.5% was associated with an increased risk of adverse pregnancy outcomes, including pre-eclampsia, preterm birth, and LGA infants [20]. A large prospective nationwide birth cohort study from Japan with HbA1c < 6.5% (< 48 mmol/mol) reported that every 1% (11 mmol/mol) increase in HbA1c levels measured less than 24 weeks of gestation, were directly associated with a higher risk of adverse pregnancy outcomes [21]. Similarly, another study by Mane et al., in a multiethnic community reported that an early HbA1c level of 5.9%, unrelated to GDM, indicated an increased risk for macrosomia [22].

Our results require cautious interpretation since the HbA1c categories used in our study were developed by ADA for the diagnosis of diabetes in non-pregnant individuals. In women with pre-existing diabetes, early pregnancy HbA1c levels directly correlate with pregnancy outcomes [22–24], but this association is still ambiguous in those without diabetes. It is also important to note that the predictive value of HbA1c for adverse outcomes may vary depending on factors such the timing of HbA1c measurement during pregnancy. Carlsen et al. examined the association between HbA1c levels measured during mid-pregnancy and adverse outcomes in women with pre-existing diabetes. The study found that HbA1c levels in the upper quartile (but still within the generally accepted normal range) are at increased risk of preterm delivery and preeclampsia [25]. Similarly, Hong et al. investigated that predelivery HbA1c at term in a healthy pregnant population is a potential predictor for adverse pregnancy outcomes such as c-section deliveries [26]. Nielsen et al. found that HbA1c was lower in early pregnancy and further decreased in late pregnancy compared with age-matched nonpregnant women. A decrease of the upper normal limit of HbA1c from 6.3% before pregnancy to 5.6% in the third trimester of pregnancy was of significant clinical importance [27]. Thus, it may not be possible to compare the studies performed at different time points during pregnancy.

The relationship between HbA1c levels and adverse pregnancy outcomes also varies by ethnicity. Results from a multiethnic cohort study in Barcelona, corresponded to a significant association between high normal range HbA1c and the risk of macrosomia, but no associations between the HbA1c level with preterm birth and LGA could be established after adjustment of potential confounders [22]. These results could in part be attributed to the differences in ethnic origins of the study populations. The research by Hughes et al*.* was conducted in a relatively low-risk, predominantly white population, whereas the cohort in the current study was characterized by an entirely south Asian or African population hailing from very different socio-economic settings and health conditions [18]. Previous studies reported an interracial variability in HbA1c levels and in pregnancy outcomes [23, 28].

Studies indicate that 70–85% of women diagnosed with GDM according to Carpenter-Coustan or National Diabetes Data Group (NDDG) criteria can effectively control GDM through lifestyle modifications alone and some pregnant women with hyperglycemia may require frequent glucose testing and continuous use of either oral or injectable medications [29–31]. Thus, adopting the early to mid-pregnancy point-of-care test HbA1c test in community-settings can enable timely management and targeted pregnancy-focused education for future risk reduction. The costs of identifying a greater number of the pregnant population to be at an increased risk of adverse outcomes could be balanced by the efficient management of the hyperglycemia and avoidance of consequent healthcare costs associated with an LGA or preterm delivery.

We studied a large population-based cohort of women in a setting with universal HbA1c testing in early pregnancy, minimizing the potential for selection bias. We used standardized cut-offs which may cause ease of comparison with other studies. Our study adjusted for systemically identified confounders in a general pregnant population. Our population was well defined, and our sample size appropriately calculated for a multivariable binary logistic regression analysis. Our study population was multiethnic, and we believed our results would be generalizable to a similar population and care setting.

Our study has some limitations. The ADA defined cut-offs were developed specifically for diagnosing diabetes and monitoring blood glucose control in diabetic patients, not for predicting pregnancy outcomes in healthy pregnant women. Therefore, it is possible that these cut-offs may not be optimal for predicting pregnancy outcomes in this population. Although we carefully adjusted for potential confounders, we are unlikely to completely rule out the possibility of vestigial confounding by other undocumented determinants, such as family history of diabetes, diagnosis of GDM in a prior pregnancy, gestational weight gain, dietary nutrition during gestation, and other socioeconomic parameters. Additionally, it is also widely recognized that hemoglobinopathies are more frequent in some nonwhite populations and that their presence might influence HbA1c levels [32]. Furthermore, in this study, pregnant women were not screened for GDM, which meant that it could not be included as a confounder and so an expected influence on results is a rational possibility.

## Conclusion

In conclusion, maternal HbA1c level is an independent risk factor for predicting adverse pregnancy outcomes such as stillbirth, preterm birth, and LGA among women in South Asia and Sub-Saharan Africa. These groups may benefit from early interventional strategies. Further research is required to predict the diagnostic accuracy of the test as compared to the gold standard in these settings.

### Supplementary Information

**Additional file 1:** **Figure S1. **Mean Hba1c levels site-wise. **Figure S2.** HbA1c Levels by (a) Maternal Age, (b) BMI and (c) MUAC.

## Data Availability

Data are available from the corresponding author upon reasonable request.
