# Supplementary material for: Early to mid-pregnancy HbA1c levels and its association with adverse pregnancy outcomes in three low middle-income countries in Asia and Sub-Saharan Africa
Source: BMC Pregnancy Childbirth. 2024 Jan 15;24:66. doi: 10.1186/s12884-023-06241-w (PMC10789021; doi:10.1186/s12884-023-06241-w)
Supplement: Supplementary file 1 — Additional file 1: Figure S1. Mean Hba1c levels site-wise. Figure S2. HbA1c Levels by (a) Maternal Age, (b) BMI and (c) MUAC. [file 12884_2023_6241_MOESM1_ESM.docx]

**Figure S1 : Mean Hba1c levels site-wise**

**Figure s2:** **HbA1c Levels by (a) Maternal Age, (b) BMI and (c) MUAC.**
